# Bio-inspired computational heuristics to study Lane–Emden systems arising in astrophysics model

**DOI:** 10.1186/s40064-016-3517-2

**Published:** 2016-10-24

**Authors:** Iftikhar Ahmad, Muhammad Asif Zahoor Raja, Muhammad Bilal, Farooq Ashraf

**Affiliations:** 1 Department of Mathematics, University of Gujrat, Gujrat, 50700 Pakistan; 2 Department of Electrical Engineering, COMSATS Institute of Information Technology, Attock, 43600 Pakistan; 3 Faculty of Science and Technology, University of Malaysia Pahang, Pekan, Pahang Malaysia

**Keywords:** Singular systems, Computational intelligence, Genetic algorithm, Artificial neural networks, Pattern search methods, Sequential quadratic programming

## Abstract

This study reports novel hybrid computational methods for the solutions of nonlinear singular Lane–Emden type differential equation arising in astrophysics models by exploiting the strength of unsupervised neural network models and stochastic optimization techniques. In the scheme the neural network, sub-part of large field called soft computing, is exploited for modelling of the equation in an unsupervised manner. The proposed approximated solutions of higher order ordinary differential equation are calculated with the weights of neural networks trained with genetic algorithm, and pattern search hybrid with sequential quadratic programming for rapid local convergence. The results of proposed solvers for solving the nonlinear singular systems are in good agreements with the standard solutions. Accuracy and convergence the design schemes are demonstrated by the results of statistical performance measures based on the sufficient large number of independent runs.

## Background

### Introduction

Mathematical models based on Lane–Emden type equations (LEEs) have been studied in diverse fields of applied sciences, particularly, in the domain of astrophysics. Singular second order nonlinear initial value problem (IVP) of LEEs describes various real life phenomena. Generally, the most of the problems arising in astrophysics are modelled by second order nonlinear ordinary differential equations (ODEs) (Lane 1870; Emden 1907; Fowler 1914, 1931). The general form of LFE is represented mathematically as:

$$\frac{{d^{2} y}}{{dx^{2} }} + \frac{\alpha }{x}\frac{dy}{dx}+f (x , {\text{y)}} = g(x),{\kern 1pt}$$

for $\alpha ,x \ge 0,$ having initial conditions as:

$$y(0) =a, \quad \frac{dy(0)}{dx} = 0,$$

here *a* is constant and *f*(*x*, *y*) is a nonlinear function. LEE has a singularity at the origin, i.e., *x* = 0, for the above conditions. By taking $\alpha = 2$, $f(x,y) = y^{n}$, $g(x) = 0$ and $a = 1$ in the above equations, we get

1 $$\frac{1}{{x^{2} }}\frac{d}{dx}\left( {x^{2} \frac{dy}{dx}} \right) = - y^{n} ,$$

with initial conditions as:

2 $$y(0) = 1,\quad \frac{dy(0)}{dx} = 0,$$

where *n* ≥ 0 is constant. The LEEs arise in the study of the theory of stellar structure, isothermal gas spheres, thermal behavior of a spherical cloud of gas and thermionic current models (Davis 1962; Chandrasekhar 1967; Datta 1996). Reliable and accurate solution of ODEs, with singularity behavior in various linear and nonlinear IVPs of astrophysics is a new challenge for researchers now a day.

In astrophysics, a fluid obeys a polytropic equation of state under the assumption, and then with suitable transformation laws Eq. (1) is equivalent to equation of static equilibrium. Further in case of the gravitational potential of a self-gravitating fluids the LEE is also called Poisson’s equation. Physically, hydrostatic equilibrium provides a connection between the gradient of the potential, the pressure and the density. Numerical simulation is presented in (Shukla et al. 2015) for two dimensional Sine–Gordon equation.

Analytically, it is difficult to solve these equations, so various techniques like Adomian decomposition method (ADM), differential transformation method (DTM) and perturbation temple technique based on series solutions have been used (Wazwaz 2001, 2005, 2006; Mellin et al. 1994). Ramos (2005) solved singular IVPs of ODEs using linearization procedures. Liao (2003) presented ADM for solving LEEs (Chowdhury and Hashim 2007a). Chowdhury and Hashim (2007b) employed Homotopy-perturbation method (HPM) to get the solution for singular IVPs of LEEs. Dehghan and Shakeri (2008) provides the solution of ODEs models arising in the astrophysics field using the variational iteration procedure. Further, the solution of Emden–Fowler equation (EFE) is also reported by incorporating the method of Lie and Painleve analysis by Govinder and Leach (2007). Kusano provide solutions for nonlinear ODEs based on EFEs (Kusano and Manojlovic 2011). Muatjetjeja and Khalique (2001) given the exact solution for the generalized LEEs of two kinds. Modified Homotopy analysis method (HAM) is used by Singh et al. (2009) and Mellin et al. (1994) to get the numerical solution of LEEs. Demir and Sungu (2009) gives the numerical solutions of nonlinear singular IVP of EFEs using DTM. Shukla et al. (2015) provides the studies using the cubic B-spline differential quadrature method. Moreover, neural networks applications in astronomy, Astrophysics and Space Science can be seen in Bora et al. (2008, 2009), Bazarghan and Gupta (2008), Singh et al. (1998, 2006), Gupta et al. (2004), Gulati et al. (994).

Recently, a lot of effects has been made by the researcher in the field of artificial neural networks (ANNs) to investigate the solution of the IVPs and boundary value problems (BVP) (Ahmad and Bilal 2014; Rudd and Ferrari 2015; Raja 2014a; Raja et al. 2015b). Well-established strength of neural networks as a universal function approximation optimized with local and global search methodologies has been exploited to solve the linear and nonlinear differential equations such as problems arising in nanotechnology (Raja et al. 2016b, e), fluid dynamics problems based on thin film flow (Raja et al. 2015a, 2016d), electromagnetic theory (Khan et al. 2015), fuel ignition model of combustion theory (Raja 2014b), plasma physics problems based on nonlinear Troesch’s system (Raja 2014c), electrical conducting solids (Raja et al. 2016c), magnetohydrodynamic problems (Raja and Samar 2014e) Jaffery-Hamel flow in the presence of high magnetic fields (Raja et al. 2015c), nonlinear Pantograph systems (Ahmad and Mukhtar 2015; Raja 2014d; Raja et al. 2016a) and many others. These are motivating factors for authors to develop a new ANNs based solution of differential equations, which has numerous advantages over its counterpart traditional deterministic numerical solvers. First of all, ANN methodologies provide the continuous solution for the entire domain of integration, generalized method which can be applied for the solution of other similar linear and nonlinear singular IVPs and BVPs. Aim of the present research is to develop the accurate, alternate, robust and reliable stochastic numerical solvers to solve the Lane-Enden equation arising in astrophysics models.

Organization of the paper is as follows: “Methods” section gives the proposed mathematical modelling of the system. In “Learning methodologies” section, learning methodologies are presented. Numerical experimentation based on three problems and cases is presented in “Results and discussion” section. In “Comparison through statistics” section comparative studies and statistical analysis are presented. In last section conclusions is drawn with future research directions.

## Methods

### Mathematical modelling

In this section, differential equation neural networks mathematical modelling of LEEs has been given. Arbitrary combine feed-forward ANNs are used to model the equation, while. Log-sigmoid based transfer function is used in the hidden layer of the networks. Following continuous mapping representing the solution *y*(*x*) and its respectively derivations is used to solve LEFs (Raja et al. 2016e; Raja 2014c).

3 $$\left\{ \begin{aligned} &\hat{y}\left( x \right) = \mathop \sum \limits_{i = 1}^{m} \delta_{i} f\left( {\beta_{i} + \omega_{i} x} \right) \hfill \\ &\frac{{d\hat{y}\left( x \right)}}{dx} = \mathop \sum \limits_{i = 1}^{m} \delta_{i } \frac{d}{dx}f\left( {\beta_{i} + \omega_{i} x} \right) \hfill \\ &\vdots \hfill \\ &\frac{{d^{n} \hat{y}\left( x \right)}}{{dx^{n} }} = \mathop \sum \limits_{i = 1}^{m} \delta_{i } \frac{{d^{n} }}{{dx^{n} }}f\left( {\beta_{i} + \omega_{i} x} \right) \hfill \\ \end{aligned} \right.,$$

where the ‘hat’ on the top of the symbol *y*(*x*) denotes their estimated values while *δ*_*i*_, *β*_*i*_, and *ω*_*i*_ are bounded real-valued representing the weights of the ANN models for *m* the number of neurons and *f* is the activation function, which is equal to:

$$f(x) = \frac{1}{{1 + e^{ - x} }}$$

for the hidden layers of the networks.

Using log-sigmoid, ANNs based approximation of the solution and few of its derivatives can be written as:

4 $$\left\{ \begin{aligned} &\hat{y}_{LS} \left( x \right) = \mathop \sum \limits_{i = 1}^{m} \frac{{\delta_{i} }}{{1 + e^{{( - \beta_{i} - \omega_{i} x)}} }} \hfill \\ &\hat{y}_{LS}^{\left( 1 \right)} \left( x \right) = \mathop \sum \limits_{i = 1}^{m} \frac{{\delta_{i} \omega_{i} e^{{( - \beta_{i} - \omega_{i} x)}} }}{{[1 + e^{{( - \beta_{i} - \omega_{i} x)}} ]^{2} }}{\kern 1pt} \hfill \\ &\hat{y}_{LS}^{\left( 2 \right)} \left( x \right) = \mathop \sum \limits_{i = 1}^{m} \delta_{i} \omega_{i}^{2} \left[ {\frac{{2e^{{( - 2\beta_{i} - 2\omega_{i} x)}} }}{{[1 + e^{{( - \beta_{i} - \omega_{i} x)}} ]^{3} }} - \frac{{e^{{\left( { - \beta_{i} - \omega_{i} x} \right)}} }}{{[1 + e^{{\left( { - \beta_{i} - \omega_{i} x} \right)}} ]^{2} }}} \right] \hfill \\ \end{aligned} \right.$$

where $\hat{y}_{LS}^{\left( 1 \right)} \left( {\text{x}} \right)$ and $\hat{y}_{LS}^{\left( 2 \right)} \left( {\text{x}} \right)$ represent the first and second order derivative with respect to *x*.

In Eq. (1), the generic form of nonlinear singular Lane–Emden equation is given. While in Eqs. (3–5) continuous mapping of neural networks models for approximate solution $\hat{y}\left( x \right)$ and its derivatives are presented in term of single input, hidden and output layers. Additionally, in the hidden layers log-sigmoid activation function and its derivatives are used for y(t) and its derivatives, respectively.

### Fitness function formulation

The objective function or fitness function is formulated by defining an unsupervised manner of differential equation networks (DEN) of given equation and its associated boundary conditions. It is defined as, where the mean square error term $\in_{1}$ is associated with Eq. (1) is given as (Raja 2014c, d):

5 $$\in_{1} = \frac{1}{N + 1}\mathop \sum \limits_{k = 0}^{N} \left( {\frac{d}{dx}\left( {x^{2}_{k} \frac{{d\hat{y}_{k} }}{dx}} \right) + x_{k}^{2} \hat{y}_{k}^{n} } \right)^{2} ,$$

where $$\hat{y}_{k} = \hat{y}\left( {x_{k} } \right), N = \frac{1}{h} ,x_{k} = kh(k = 0,1,2, \ldots ,N).$$

The interval is divided into number of steps with step size and mean square error for Eq. (2) can be defined as follows:

6 $$\in_{2} \,= \frac{1}{2}\left[ {\left( {\hat{y}\left( 0 \right) - 1} \right)^{2} + \left( {\frac{{d\hat{y}\left( 0 \right)}}{dx} - 0} \right)^{2} } \right]$$

By combining Eqs. (6) and (7), we obtain the fitness function

7 $$\in \,= \frac{1}{N + 1}\mathop \sum \limits_{k = 0}^{N} \left( {\frac{d}{dx}\left( {x^{2}_{k} \frac{{d\hat{y}_{k} }}{dx}} \right) + x_{k}^{2} \hat{y}_{k}^{n} } \right)^{2} + \frac{1}{2}\left[ {\left( {\hat{y}\left( 0 \right) - 1} \right)^{2} + \left( {\frac{{d\hat{y}\left( 0 \right)}}{dx} - 0} \right)^{2} } \right].$$

**Fig. 1:**
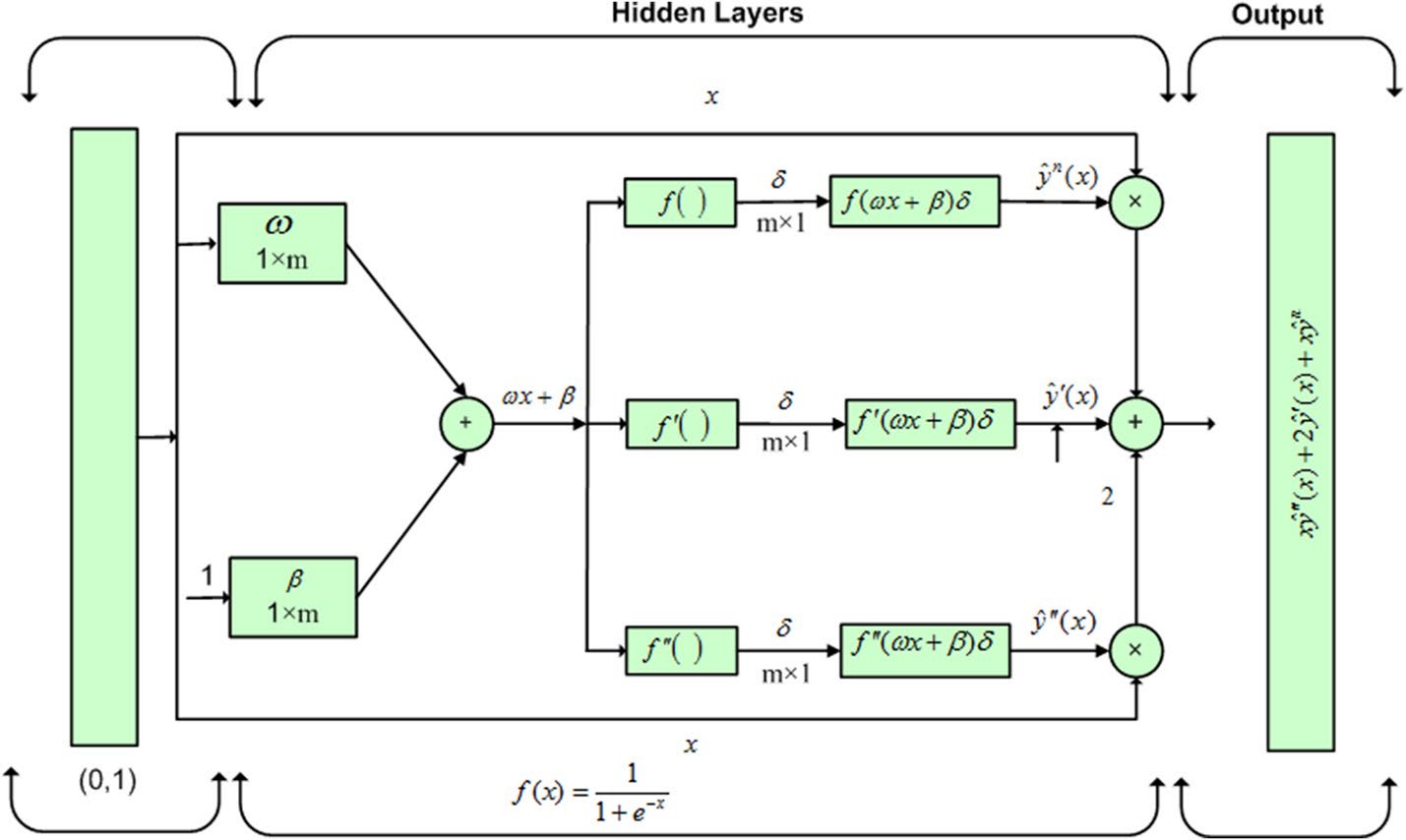
Design of neural network architecture for nonlinear singular Lane–Emden type equation

The ANNs architecture for nonlinear singular LEEs is presented in Fig. 1.

## Learning methodologies

*Pattern search (PS)* optimization technique belongs to a class of direct search methods, i.e., derivative free techniques, which are suitable to solve effectively the variety of constrained and unconstrained optimization problems. Hooke et al., are the first to introduce the name of PS technique (Hooke and Jeeves 1996), while the convergence of the algorithm was first proven by Yu (1979). In standard operation of PS technique, a sequence of points, which are adapted to desire solution, is calculated. In each cycle, the scheme finds a set of points, i.e., meshes, around the desired solution of the previous cycle (Dolan et al. 2003). The mesh is formulated by including the current point multiplied by a set of vectors named as a pattern (Lewis et al. 1999). PS technique is very helpful to get the solution of optimization problem such as minimization subjected to bound constrained, linearly constrained and augmented convergent Lagrangian algorithm (Lewis et al. 2000).

Genetic algorithms (GAs) belong to a class of bio-inspired heuristics develop on the basis of mathematical model of genetic processes (Man et al. 1996). GAs works through its reproduction operators based on selection operation, crossover techniques and mutation mechanism to find appropriate solution of the problem by manipulating candidate solutions from entire search space (Cantu-Paz 2000). The candidate solutions are generally given as strings which is known as chromosomes. The entries of chromosome are represented by genes and the values of these genes represents the design variables of the optimization problem. A set of chromosome in GAs is called a population which used thoroughly in the search process. A population of few chromosomes may suffer a premature convergence where as large population required extensive computing efforts. Further details, applications and recent trends can be seen in (Hercock 2003; Zhang et al. 2014; Xu et al. 2013).

*Sequential quadratic programming (SQP)* belong to a class of nonlinear programming techniques. Supremacy of SQP method is well known on the basis of its efficiency and accuracy over a large number of benchmark constrained and unconstrained optimization problems. The detailed overview and necessary mathematical background are given by Nocedal and Wright (1999). The SQP technique has been implemented in numerous applications and a few recently reported articles can be seen in Sivasubramani et al. (2011), Aleem and Eldeen (2012).

**Fig. 2:**
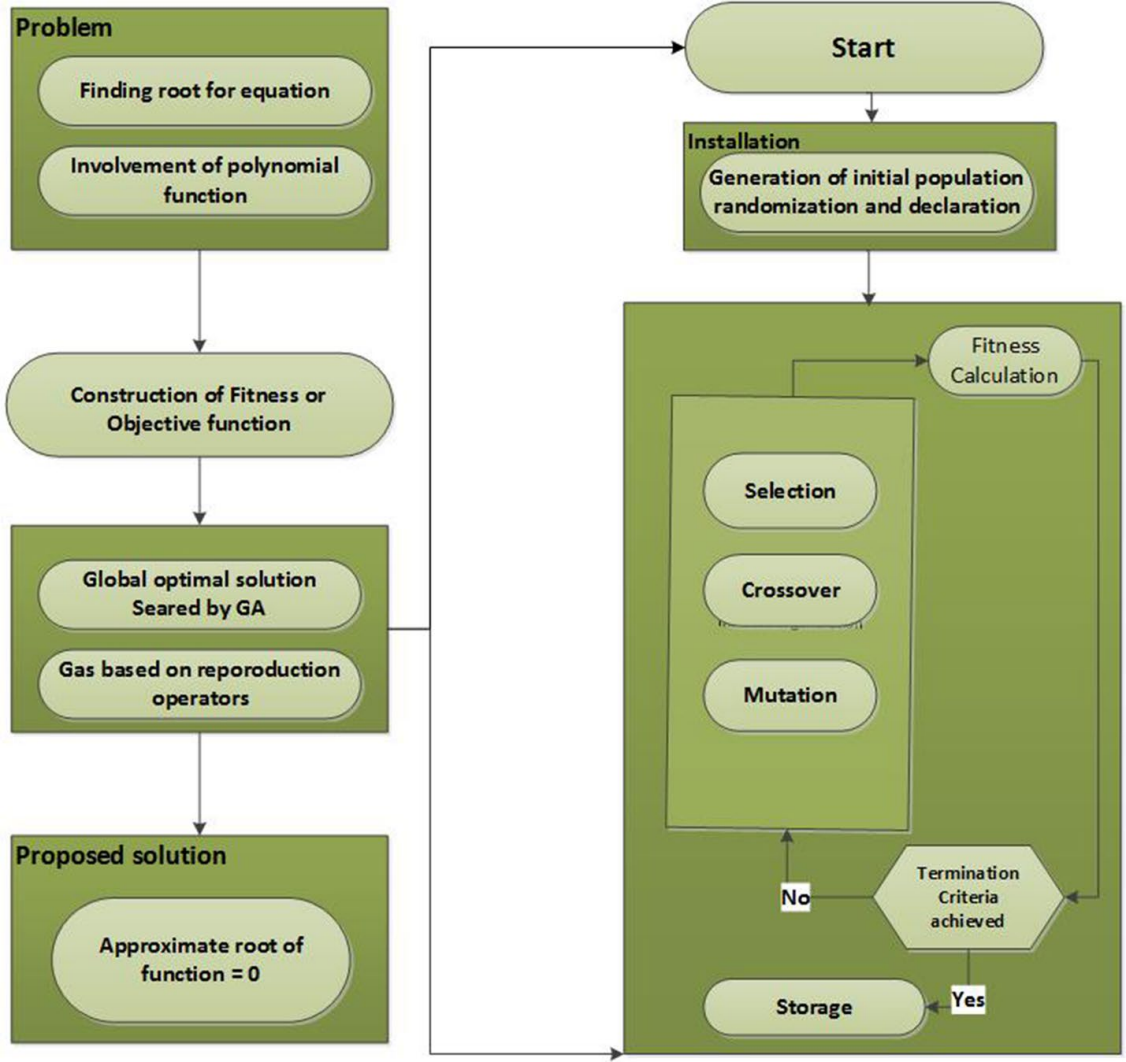
Flow chart of genetic algorithm

In simulation studies, we have used MATLAB optimization toolbox for running of SQP, PS, and GAs as well as hybrid approaches based on PS-SQP and GA-SQP to get the suitable parameters of ANN models. The workflow diagram of the proposed methodology base on GA-SQP to get the appropriate design parameters is shown in Fig. 2 while the procedure of GA-SQP to find the optimized weight vector of ANN is given below:

- **Step 1:** *Initialization:* create an initial population with bounded real numbers with each row vector represents chromosomes or individuals. Each chromosome has number of genes equal to a number of design parameters in ANN models. The parameter settings GAs are given in Table 1.

**Table 1:** Parameters setting for SQP, PS and GA respectively

Methods	Parameter	Setting	Parameter	Setting
SQP	Initial weight vector creation	Randomly between (−1, 1)	Maximum function evaluation	10000
	Number of variable	30	Fitness limit	10^−25^
	Total initial weight vectors	1000	X tolerance	10^−25^
	Number of iterations	10000	Function tolerance	10^−30^
	Derivative	By solvers	Nonlinear constraint tolerance	10^−30^
	Finite difference type	Central	Upper bound	−10
	Hessian	BFGS	Lower bound	10
	Algorithm	SQP	Others	Default
PS	Solver	Pattern search	Maximum size	Inf
	Start point	Randn (1, 30)	Scale	On
	Poll method	GPS positive 2N	Bind tolerance	10^−03^
	Complete poll	Off	Maximum iteration	2000
	Polling order	Consecutive	Max function evaluation	1,000,000
	Initial size	1	X tolerance	10^−25^
	Expansion factor	2	Function tolerance	10^−30^
	Tolerance	Eps	Nonlinear constraint tolerance	10^−30^
	Mesh tolerance	10^−32^	Plot	Function value
GA	Solver	GA	Pop type	Double vector
	No of variables	30	Pop size	[30, 30, 30, 30, 30, 30, 30, 30, 30]
	Time limit	Default	Initial range	[0,1]
	Generations	1,000,000	Scaling fun	Rank
	Stall generations	2000	Selection fun	Stochastic
	Function tolerance	10^−28^	Interval	20
	Nonlinear constraint tolerance	10^−28^	Fraction	0.2
	Initial penalty	10	Plot	Best function
	Penalty factor	100	Elite count	2
	Crossover	Forward	Time limit	Inf
	Direction	Forward	Other	Defaults
- **Step 2:** *Fitness evaluations:* Determined the value of the fitness row vector of the population using the Eq. (7).
- **Step 3:** *Stoppage:* Terminate GAs on the basis of following criteria.
- The predefined fitness value $\in$ is achieved by the algorithm, i.e., $\in < 10^{ - 15} .$
- Total number of generations/iterations are executed.
- Any of the termination conditions given in Table 1 for GAs are achieved.

If any of the stopping criteria is satisfied, then go to step 6 otherwise continue

- **Step 4:** *Ranking:* Rank each chromosome on the basis minimum fitness $\in$ values. The chromosome ranked high has small values of the fitness and vice versa.
- **Step 5:** *Reproduction:* Update population for each cycle using reproduction operators based on crossover, mutation selection and elitism operations. Necessary settings of these fundamental operators are given in Table 1.

Go to step 2 with newly created population

- **Step 6:** *Refinement:* The Local search algorithm based on SQP is used for refinement of design parameters of ANN model. The values of global best chromosome of GA are given to SQP technique as an initial start weight. In order to execute the SQP algorithm, we incorporated MATLAB build in function ‘*fmincon*’ with algorithm SQP. Necessary parameter settings for SQP algorithm is given in Table 1.
- **Step 7:** *Data Generation and analysis:* Store the global best individual of GA and GA-SQP algorithm for the present run. Repeat the steps 2–6 for multiple independent runs to generate a large data set so that reliable and effective statistical analysis can be performed.

## Results and discussion

The results of simulation studies are presented here to solve LEEs by the proposed ANN solver. Proposed results are compared with reported analytical as well as numerical methods.

### Problem I: (Case I: *n* = 0)

For *n* = 0, the Eq. (1) becomes linear ordinary differential equation, can be written as:

8 $$\frac{1}{{x^{2} }}\frac{d}{dx}\left( {x^{2} \frac{dy}{dx}} \right) + 1 = 0,$$

and its associated conditions are

9 $$y\left( 0 \right) = 1,\quad \frac{dy\left( 0 \right)}{dx} = 0.$$

The system given in Eqs. (8–9) is solved by taking 10 neuron in ANN model and resultant 30 unknown weights $\delta_{i} ,\beta_{i}$, and ${\kern 1pt} \omega_{i}$ for I = 1, 2,…, 10. The fitness function is developed for this case by taking step size *h* = 0.1 and hence 11 numbers of grid points in the interval (0, 1) as:

10 $$\in \,= \frac{1}{11}\mathop \sum \limits_{k = 0}^{10} \left[ {\frac{d}{dx}\left( {x_{k}^{2} \frac{{d\hat{y}_{k} }}{dx}} \right) + x_{k}^{2} } \right]^{2} + \frac{1}{2}\left[ {\left( {y\left( 0 \right) - 1} \right)^{2} + \left( {\frac{dy\left( 0 \right)}{dx} - 0} \right)^{2} } \right]$$

**Fig. 3:**
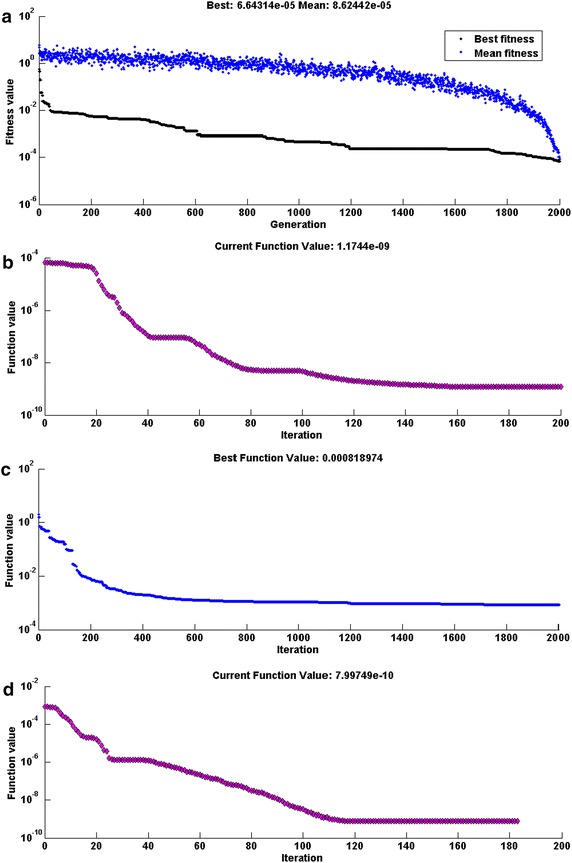
Learning curves for different optimization algorithms for Problem I. **a** GA, **b** GA-SQP, **c** PS, **d** PS-SQP

We have to find the weights for each model such that $\in \,\to 0.$ The optimization of these weights is carried out using global and local approach of algorithm with parameter setting shown in Table 1. Training of weights is carried out by SQP, PS, GA, and also with a hybrid approach of PS-SQP and GA-SQP algorithms. Parameter setting given in Table 1 will be used for these algorithms. These algorithms are run and weights are trained through the function of algorithms; one specific set of weights learned by SQP, PS, GA, PS-*SQP*, *GA*-*SQP algorithms yield fitness* values of 1.90*E*−09, 4.97*E*−07, 6.45*E*−07, 5.60*E*−10, 9.38*E*−09, respectively, are given in Table 2. While the learning plots based on fitness against number of iterations for GA, GA-SQP, PS and PS-SQP algorithm are given Fig. 3.

**Table 2:** Best weights trained for neural network modelling by SQP, PS and PSO-SQP algorithms

Methods	*I*	$\delta_{i}$	*β*_*i*_	_*ωi*_	*i*	*δ* *i*_*i*_	*β*_*i*_	_*ωi*_
SQP	1	0.78636	0.11707	−1.72866	6	2.24414	−1.07067	3.55435
	2	−2.80683	0.84866	−1.47352	7	0.72638	0.80491	−1.05825
	3	−0.66561	0.11462	−1.56349	8	−0.35824	−0.30248	−0.81978
	4	−2.77591	−0.10056	−0.56814	9	−0.93043	−0.67351	0.00426
	5	0.35878	−1.54018	−0.39116	10	0.77053	1.44174	1.15517
PS	1	3.03789	−0.35385	0.02289	6	−1.38575	4.00223	−8.80831
	2	0.82521	−0.82359	1.73409	7	1.10965	−0.74481	−1.14468
	3	−8.99993	−3.18106	−4.91916	8	−0.27787	−3.01028	0.61488
	4	−1.05818	0.54020	1.74266	9	−5.93029	0.35017	−2.00264
	5	7.54212	0.28198	−0.83137	10	−2.05182	0.45484	2.87243
PS-SQP	1	−0.72218	0.55533	3.06589	6	−1.26715	1.30549	−1.92557
	2	0.70591	3.76635	4.43837	7	−1.18046	−1.15109	−0.18455
	3	0.09391	3.18733	1.15390	8	0.00790	1.08650	0.35001
	4	7.63559	−0.07725	−1.55487	9	0.06668	−4.96006	7.78676
	5	1.38422	−0.76144	−1.69561	10	0.00121	−6.38647	6.67553
GA	1	1.29680	−0.70586	0.94321	6	0.18766	2.70748	−1.62678
	2	−1.08867	0.43791	1.16502	7	0.31812	2.33138	−0.61683
	3	0.95837	−0.89643	1.47983	8	−0.06982	0.73937	−0.09366
	4	0.415759	3.43301	1.48746	9	−0.55372	0.98114	1.17121
	5	−0.01698	5.29995	−2.90769	10	0.14876	1.78333	0.46163
GA-SQP	1	−0.17494	1.78344	0.98665	6	−0.19778	0.02527	0.14497
	2	−0.91882	−0.05487	0.84314	7	−0.18451	−0.15034	0.50728
	3	−0.07822	0.34068	0.50753	8	0.32558	−1.24882	2.01797
	4	0.75215	1.77652	1.46356	9	2.07449	−1.21908	3.30059
	5	−0.08314	1.50680	−1.73595	10	−1.08201	0.038511	1.28033

Results of learning plots shows that rate of convergence (reduction in the fitness values) and accuracy of GA is relatively better than that of PS techniques, while, the results of the hybrid approaches i.e., PS-SQP and GA-SQP, are found better than that of GA and PS techniques. Additionally, very small difference is observed between the results of the hybrid approaches, however GA-PS results are a bit superior.

**Fig. 4:**
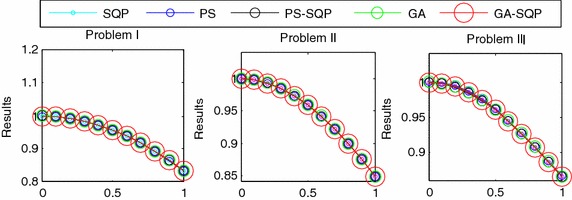
The graph between the approximated result and reported results of Problems I, II and III

Proposed results have been compared with the analytical results (Davis 1962) and numerical technique (Mall and Chakraverty 2014) which have been presented in Table 3. Mean Absolute errors (MAE) reported in SQP, PS, GA, PS-SQP, GA-SQP algorithms are 5.44*E*−07, 3.18*E*−07, 1.51*E*−06, 9.03*E*−06, 6.67*E*−07 as given in Table 4. Mean square error (MSE) reported in SQP, PS, GA, PS-SQP, GA-SQP algorithms are 3.35*E*−06, 2.95*E*−05, 2.73*E*−04, 1.65*E*−04, 5.58*E*−06 for data presented in Table 4. Further, a graphical representation of proposed results for these problems is shown in Fig. 4. After comparison of proposed solution with reported results of Mall and Chakraverty (2014), PS-SQP gives the absolute error 5.60*E*−10 while the absolute error of Mall and Chakraverty (2014) is 1.00*E*−03 which definitely shows the better convergence of the proposed method. The mean absolute error (MAE) is defined as:

**Table 3:** Comparative studies of the results of proposed methodologies for Problem I

X	Exact	Reported solution		Proposed approximated solution $\hat{y}({\text{x}})$				
	y(x)	Analytical result	ChNN (Mall and Chakraverty 2014)	SQP	PS	GA	PS-SQP	GA-SQP
0	1.000000	1.0000	1.0000	1.000000	1.000001	1.000037	1.000000	1.000000
0.1	0.998333	0.9983	0.9993	0.998349	0.998322	0.998451	0.998224	0.998339
0.2	0.993333	0.9933	0.9901	0.993376	0.993513	0.993512	0.993197	0.993341
0.3	0.985000	0.9850	0.9822	0.985053	0.985395	0.985151	0.984859	0.985006
0.4	0.973333	0.9733	0.9766	0.973380	0.973868	0.973433	0.973186	0.973341
0.5	0.958333	0.9583	0.9602	0.958369	0.958901	0.958420	0.958184	0.958341
0.6	0.940000	0.9400	0.9454	0.940032	0.940514	0.940122	0.939851	0.940007
0.7	0.918333	0.9183	0.9134	0.918369	0.918761	0.918511	0.918183	0.918340
0.8	0.893333	0.8933	0.8892	0.893375	0.893700	0.893543	0.893182	0.893340
0.9	0.865000	0.8650	0.8633	0.865044	0.865370	0.865192	0.864848	0.865007
1	0.833333	0.8333	0.8322	0.833373	0.833750	0.833480	0.833181	0.833340

**Table 4:** Comparative studies based on values of absolute errors (AE) for Problem I

X	Proposed methods (AE)					Reported (AE)
	SQP	PS	GA	PS-SQP	GA-SQP	ChNN (Mall and Chakraverty 2014)
0	1.90E−09	4.97E−07	6.45E−07	5.60E−10	9.38E−09	0.00E+00
0.1	1.47E−06	3.47E−05	2.72E−05	1.03E−07	2.07E−06	1.00E−03
0.2	4.35E−06	8.80E−05	1.41E−05	3.60E−07	3.51E−06	3.20E−03
0.3	5.61E−06	1.03E−04	8.75E−05	4.93E−07	2.58E−06	2.80E−03
0.4	5.16E−06	8.88E−05	1.33E−04	4.67E−07	2.19E−06	3.30E−03
0.5	4.09E−06	7.04E−05	1.26E−04	3.73E−07	2.95E−06	1.90E−03
0.6	3.47E−06	6.62E−05	8.39E−05	3.12E−07	3.24E−06	5.40E−03
0.7	3.71E−06	7.61E−05	3.88E−05	3.12E−07	2.62E−06	4.40E−03
0.8	4.39E−06	8.77E−05	2.09E−05	3.50E−07	2.42E−06	4.10E−03
0.9	4.69E−06	8.86E−05	4.08E−05	3.78E−07	2.99E−06	1.70E−03
1	4.27E−06	8.01E−05	7.66E−05	3.46E−07	2.69E−06	1.10E−03

$$MAE = AVERAGE_{(i = 1,2,3, \ldots 11)} \left| {y\left( {x_{i} } \right) - \hat{y}(x_{i} )} \right|$$

The MAE values for SQP, PS, GA, PS-SQP, GA-SQP algorithms are evaluated as 1.01*E*−05, 4.09*E*−05, 4.09*E*−04, 3.40*E*−04, and 9.15*E*−06, respectively. The results of statistical analysis based on 100 independent runs of each algorithm for input with step size of 0.1 is given in Table 5. Smaller values of statistical performance indices verified the consistent correctness of the proposed schemes.

**Table 5:** Results of statistics based on 100 independent runs of each algorithm

Methods	*x*	Min	Max	Mean	Median	STD	Variance	MSE
SQP	0.1	2.06E−09	1.58E−05	3.35E−06	2.71E−06	2.81E−06	7.92E−12	3.35E−06
	0.3	3.54E−07	7.33E−05	1.39E−05	1.10E−05	1.19E−05	1.41E−10	1.39E−05
	0.5	3.19E−08	1.03E−04	1.23E−05	8.93E−06	1.32E−05	1.74E−10	1.23E−05
	0.7	3.07E−07	8.17E−05	1.06E−05	7.53E−06	1.07E−05	1.15E−10	1.06E−05
	0.9	4.25E−07	7.35E−05	1.19E−05	9.49E−06	1.08E−05	1.16E−10	1.19E−05
PS	0.1	8.94E−07	1.24E−03	2.73E−04	2.08E−04	4.82E−05	2.32E−09	2.95E−05
	0.3	1.71E−06	1.52E−03	4.66E−04	3.66E−04	1.09E−04	1.20E−08	4.73E−05
	0.5	2.67E−06	1.39E−03	4.76E−04	3.61E−04	1.06E−04	1.12E−08	4.74E−05
	0.7	3.82E−07	1.56E−03	4.52E−04	3.65E−04	8.49E−05	7.20E−09	4.54E−05
	0.9	1.56E−06	1.52E−03	4.65E−04	3.42E−04	1.01E−04	1.01E−08	4.74E−05
PS-SQP	0.1	7.20E−08	3.51E−04	2.95E−05	1.14E−05	2.65E−04	7.02E−08	2.73E−04
	0.3	3.38E−07	9.57E−04	4.73E−05	1.72E−05	3.78E−04	1.43E−07	4.65E−04
	0.5	3.73E−07	8.88E−04	4.74E−05	1.66E−05	3.87E−04	1.50E−07	4.76E−04
	0.7	2.18E−07	6.01E−04	4.54E−05	1.81E−05	3.72E−04	1.39E−07	4.52E−04
	0.9	1.37E−07	8.15E−04	4.74E−05	1.70E−05	3.86E−04	1.49E−07	4.65E−04
GA	0.1	1.34E−07	1.55E−03	1.65E−04	7.79E−05	2.54E−04	6.45E−08	1.65E−04
	0.3	1.75E−07	2.86E−03	3.20E−04	2.13E−04	3.83E−04	1.47E−07	3.20E−04
	0.5	1.79E−07	5.57E−03	4.23E−04	1.66E−04	7.39E−04	5.46E−07	4.23E−04
	0.7	1.07E−05	5.82E−03	4.59E−04	1.52E−04	8.02E−04	6.43E−07	4.59E−04
	0.9	1.39E−06	4.39E−03	3.85E−04	1.67E−04	6.13E−04	3.76E−07	3.85E−04
GA-SQP	0.1	2.42E−07	3.68E−05	5.58E−06	4.46E−06	2.54E−04	6.45E−08	5.58E−06
	0.3	4.71E−08	5.00E−05	1.19E−05	7.77E−06	3.83E−04	1.47E−07	1.18E−05
	0.5	1.01E−07	5.00E−05	9.80E−06	7.51E−06	7.39E−04	5.46E−07	9.80E−06
	0.7	7.70E−07	5.10E−05	9.94E−06	7.26E−06	8.02E−04	6.43E−07	9.94E−06
	0.9	1.20E−07	5.21E−05	1.07E−05	7.77E−06	6.13E−04	3.76E−07	1.07E−05

### Problem II: (Case II: *n* = 1)

For *n* = 1, the Eq. (1) becomes linear ordinary differential equation of form

11 $$\frac{1}{{x^{2} }}\frac{d}{dx}\left( {x^{2} \frac{dy}{dx}} \right) + y = 0,$$

along with initial conditions given as:

12 $$y\left( 0 \right) = 1,\quad \frac{dy\left( 0 \right)}{dx} = 0.$$

The system based on Eqs. (11–12) is solved on the similar pattern of last problems and fitness function for this case is constructed as:

13 $$\in \,= \frac{1}{11}\mathop \sum \limits_{k = 0}^{10} \left[ {\frac{d}{dx}\left( {x_{k}^{2} \frac{{d\hat{y}_{k} }}{dx}} \right) + x_{k}^{2} \hat{y}_{k} } \right]^{2} + \frac{1}{2}\left[ {\left( {y\left( 0 \right) - 1} \right)^{2} + \left( {\frac{dy\left( 0 \right)}{dx} - 0} \right)^{2} } \right]$$

The algorithms are executed and weights are trained through the process. One specific set of weights learned by the SQP, PS, GA, PS -SQP, GA-SQP algorithms yield fitness values of 5.56*E*−08, 1.84*E*−07, 9.06*E*−07, 7.06*E*−09 and 4.98*E*−09 respectively. The comparison of proposed fitness results for problem II with reported results Davis (1962) and Mall and Chakraverty (2014) is provided in Table 6. While the comparison on the basis of absolute error (AE) is given in Table 7. Mean Absolute error (MAE) reported in the SQP, PS, GA, PS-SQP, GA-SQP algorithms is 3.61*E*−03, 3.52*E*−02, 3.64*E*−03, 3.86*E*−03, and 3.59*E*−03, respectively, while the Mean Square Error (MSE) these five algorithms are 1.05*E*−11, 6.86*E*−08, 2.98*E*−10, 3.45*E*−04 and 3.72*E*−11, respectively.

**Table 6:** Comparative studies of the results of proposed methodologies for Problem II

X	Exact	Reported solution		Proposed approximate solution $\hat{y}({\text{x}})$				
	y(x)	Analytical results	ChNN (Mall and Chakraverty 2014)	SQP	PS	GA	PS-SQP	GA-SQP
0	1.00000	1.00000	1.00000	1.00000	0.99998	0.99999	1.00000	0.99999
0.1	0.99800	0.99830	1.00180	0.99800	0.99805	0.99828	0.99833	0.99833
0.2	0.99300	0.99330	0.99050	0.99300	0.99278	0.99346	0.99335	0.99336
0.3	0.98500	0.98510	0.98390	0.98500	0.98470	0.98542	0.98513	0.98513
0.4	0.97300	0.97350	0.97340	0.97400	0.97343	0.97418	0.97375	0.97375
0.5	0.95800	0.95890	0.95980	0.95900	0.95891	0.95983	0.95935	0.95935
0.6	0.94100	0.94110	0.94170	0.94200	0.94169	0.94255	0.94209	0.94209
0.7	0.91800	0.92030	0.92100	0.92200	0.92187	0.92256	0.92217	0.92217
0.8	0.89300	0.89670	0.89250	0.91200	0.89943	0.90019	0.89979	0.89979
0.9	0.86500	0.87040	0.87000	0.87500	0.87489	0.87544	0.87521	0.87521
1	0.83300	0.8415	0.8431	0.84900	0.84832	0.84868	0.84865	0.84865

**Table 7:** Comparative studies based on values of absolute errors (AE) for Problem II

*X*	Proposed methods (AE)					Proposed (AE)
	SQP	PS	GA	PS-SQP	GA-SQP	ChNN (Mall and Chakraverty 2014)
0	5.56E−08	1.84E−07	9.06E−07	7.06E−09	4.98E−09	0.00E+00
0.1	3.28E−06	6.20E−06	2.49E−06	1.40E−06	5.89E−06	3.50E−03
0.2	3.08E−05	8.52E−06	2.87E−05	2.08E−05	3.41E−05	2.80E−03
0.3	1.39E−04	6.54E−06	1.36E−04	1.29E−04	1.41E−04	1.20E−03
0.4	4.25E−04	7.20E−06	4.21E−04	4.17E−04	4.26E−04	1.00E−04
0.5	1.02E−03	8.28E−06	1.02E−03	1.02E−03	1.02E−03	9.00E−04
0.6	2.09E−03	7.49E−06	2.09E−03	2.08E−03	2.10E−03	6.00E−04
0.7	3.84E−03	7.19E−06	3.84E−03	3.83E−03	3.84E−03	7.00E−04
0.8	6.47E−03	7.98E−06	6.47E−03	6.46E−03	6.46E−03	4.20E−03
0.9	1.02E−02	7.58E−06	1.02E−02	1.02E−02	1.02E−02	4.00E−04
1	1.53E−02	7.95E−06	1.53E−02	1.53E−02	1.53E−02	1.60E−03

The working of the proposed ANN models is evaluated in terms of accuracy and convergence on the basis of statistics through sufficient multiple runs rather on the single successful run of the scheme. The results and statistical analysis based on 100 runs of the algorithms in Table 8. In this case also relatively small values of statistical performance indices are obtained which show the worth of the proposed schemes.

**Table 8:** Results of statistical operators based on 100 independent runs of each algorithm for Problem II

Methods	*X*	Min	Max	Mean	Median	STD	Variance	MSE
SQP	0.1	0.00E+00	1.00E−05	0.00E+00	0.00E+00	2.43E−06	5.92E−12	1.05E−11
	0.3	0.12E−04	0.19E−02	0.14E−03	0.14E−03	9.59E−06	9.19E−11	1.92E−08
	0.5	0.99E−03	0.10E−02	0.10E−02	0.10E−02	8.71E−06	7.59E−11	1.05E−06
	0.7	0.31E−02	0.38E−02	0.38E−02	0.31E−02	6.10E−06	3.72E−11	1.47E−05
	0.9	0.10E−01	0.10E−01	0.10E−01	0.10E−01	5.88E−06	3.45E−11	1.04E−04
PS	0.1	5.35E−06	1.16E−03	2.62E−04	1.94E−04	2.40E−04	5.75E−08	6.86E−08
	0.3	6.54E−06	2.15E−03	3.46E−04	2.88E−04	3.18E−04	1.28E−07	1.20E−07
	0.5	8.28E−06	1.83E−03	8.89E−04	9.11E−04	4.08E−04	1.66E−07	7.91E−07
	0.7	7.19E−06	4.63E−03	3.67E−03	3.73E−03	5.62E−04	3.16E−07	1.34E−05
	0.9	7.58E−06	1.09E−02	9.99E−03	1.01E−02	0.11E−02	1.16E−06	9.99E−05
PS-SQP	0.1	1.66E−08	2.00E−04	1.73E−05	7.01E−06	2.65E−05	7.01E−10	2.98E−10
	0.3	8.63E−05	0.27E−02	0.14E−03	0.13E−03	2.64E−05	6.99E−10	2.09E−08
	0.5	0.74E−03	0.11E−02	0.10E−02	0.10E−02	3.65E−05	1.33E−09	1.06E−06
	0.7	0.37E−02	0.39E−02	0.38E−02	0.38E−02	3.38E−05	1.14E−09	1.48E−05
	0.9	0.10E−01	0.10E−01	0.10E−01	0.10E−02	3.05E−05	9.29E−10	0.10E−03
GA	0.1	2.04E−07	9.47E−01	0.18E−01	7.54E−05	1.18E−02	0.14E−01	0.34E−03
	0.3	0.71E−05	9.17E−02	0.18E−01	0.22E−03	1.16E−02	0.13E−01	0.33E−03
	0.5	0.36E−03	8.75E−02	0.18E−01	0.10E−02	1.13E−02	0.11E−01	0.34E−03
	0.7	0.14E−02	8.22E−02	0.20E−01	0.39E−02	1.07E−02	0.11E−01	0.40E−03
	0.9	0.5068E−02	7.57E−02	0.24E−01	0.10E−01	1.00E−02	0.10E−01	0.61E−03
GA-SQP	0.1	4.04E−08	4.27E−05	6.15E−06	4.62E−06	6.24E−06	3.89E−11	3.72E−11
	0.3	0.12E−02	0.18E−03	0.14E−03	0.14E−03	1.01E−05	1.02E−10	2.00E−08
	0.5	0.10E−02	0.10E−02	0.10E−02	0.10E−02	8.79E−06	7.72E−11	1.05E−06
	0.7	0.38E−02	0.38E−02	0.38E−02	0.38E−02	8.27E−06	6.84E−11	1.48E−05
	0.9	0.10E−01	0.10E−01	0.10E−01	0.10E−01	7.59E−06	5.76E−11	0.10E−03

### Problem III: (Case III: *n* = 5)

For *n* = 5, the Eq. (1) can be represented as:

14 $$\frac{1}{{x^{2} }}\frac{d}{dx}\left( {x^{2} \frac{dy}{dx}} \right) + y^{5} = 0,$$

with initial conditions as given below

15 $$y\left( 0 \right) = 1,\quad \frac{dy\left( 0 \right)}{dx} = 0.$$

The Eqs. (14–15) has been solved by formulating the fitness function $\in$ as:

16 $$\in \,= \frac{1}{11}\mathop \sum \limits_{k = 0}^{10} \left[ {\frac{d}{dx}\left( {x_{k}^{2} \frac{{d\hat{y}_{k} }}{dx}} \right) + x_{k}^{2} \hat{y}_{k}^{5} } \right]^{2} + \frac{1}{2}\left[ {\left( {y\left( 0 \right) - 1} \right)^{2} + \left( {\frac{dy\left( 0 \right)}{dx} - 0} \right)^{2} } \right].$$

We apply SQP, PS, GA, PS-SQP, GA-SQP algorithms to find the unknown weights of ANNs to solve Eq. (14). One set of weight for SQP, PS, GA, PS-SQP, GA-SQP algorithms with fitness vales 4.13*E*−09, 3.36*E*−06, 6.48*E*−07, 6.90*E*−06, and 6.09*E*−08, respectively, is used to obtain the solution of the equation and results are given in Tables 9 and 10. The comparison of proposed results for Problem III with reported results Davis (1962) and Mall and Chakraverty (2014) is also given in Table 9. MAE reported in the SQP, PS, GA, PS-SQP, GA-SQP algorithms is 7.91*E*−02, 9.17*E*−02, 7.80*E*−02, 7.47*E*−02 and 7.92*E*−03. MSE values reported for SQP, PS, GA, PS-SQP, GA-SQP algorithms are 6.29*E*−05, 7.05*E*−05, 8.41*E*−05, 5.58*E*−05 and 6.27*E*−05, respectively. The complete statistical analysis has been displayed in Table 11.

**Table 9:** Comparative studies of the results of proposed methodologies for Problem III

X	Exact	Reported solution		Proposed approximate solution $\hat{y}({\text{x}})$				
	y(x)	Analytical results	ChNN (Mall and Chakraverty 2014)	SQP	PS	GA	PS-SQP	GA-SQP
0	1.000	1.000	1.000	1.000	1.0013	1.0003	1.000	1.000
0.1	0.998	0.998	0.998	0.99835	1.001	0.9987	0.9983	0.99834
0.2	0.993	0.993	0.994	0.99342	0.9964	0.994	0.9935	0.99341
0.3	0.985	0.985	0.990	0.98535	0.9878	0.9861	0.9854	0.98534
0.4	0.973	0.974	0.971	0.97437	0.977	0.9751	0.9744	0.97436
0.5	0.958	0.961	0.968	0.96078	0.9635	0.9615	0.9608	0.96077
0.6	0.940	0.945	0.941	0.94492	0.9471	0.9455	0.9449	0.94492
0.7	0.918	0.927	0.930	0.92716	0.9289	0.9276	0.9271	0.92715
0.8	0.893	0.908	0.908	0.90785	0.9097	0.9082	0.9078	0.90785
0.9	0.865	0.887	0.883	0.88737	0.8888	0.8876	0.8873	0.88736
1	0.833	0.866	0.865	0.86603	0.8673	0.8662	0.866	0.86603

**Table 10:** Comparative studies based on values of absolute errors (AE) for Problem III

X	Proposed methods (AE)					Reported (AE)
	SQP	PS	GA	PS-SQP	GA-SQP	ChNN (Mall and Chakraverty 2014)
0	4.13E−09	3.361E−06	6.481E−07	6.901E−06	6.096E−08	0.00E+00
0.1	1.331E−05	5.361E−05	3.561E−05	6.251E−05	9.283E−06	2.001E−04
0.2	8.653E−05	0.171E−03	0.160E−03	0.106E−03	7.541E−05	0.123E−03
0.3	0.352E−03	0.437E−03	0.459E−03	0.113E−03	0.338E−03	0.461E−03
0.4	0.104E−02	0.114E−02	0.115E−02	0.825E−03	0.102E−02	0.324E−02
0.5	0.245E−02	0.248E−02	0.254E−02	0.228E−02	0.241E−02	0.761E−02
0.6	0.492E−02	0.497E−02	0.499E−02	0.477E−02	0.497E−02	0.384E−02
0.7	0.882E−02	0.888E−02	0.888E−02	0.867E−02	0.888E−02	0.321E−02
0.8	0.145E−01	0.145E−01	0.145E−01	0.143E−01	0.145E−01	0.234E−03
0.9	0.228E−01	0.224E−01	0.224E−01	0.222E−01	0.223E−01	0.443E−02
1	3.270E−02	3.274E−02	3.274E−02	3.259E−02	3.269E−02	9.000E−04

**Table 11:** Results of statistics based on 100 independent runs of each algorithm for Problem III

Methods	*x*	Min	Max	Mean	STD	Variance	MSE
SQP	0.1	8.691E−08	1.020E−03	2.640E−05	1.030E−04	1.070E−08	2.640E−05
	0.3	0.132E−03	0.723E−03	0.333E−03	5.730E−05	3.290E−09	3.330E−04
	0.5	0.134E−02	0.259E−02	0.242E−02	1.150E−04	1.320E−08	2.420E−03
	0.7	0.783E−02	0.893E−02	0.880E−02	1.030E−04	1.050E−08	8.800E−03
	0.9	0.218E−01	0.224E−01	0.223E−01	5.940E−05	3.530E−09	2.240E−02
PS	0.1	3.812E−06	5.934E−01	0.937E−02	0.661E−01	0.440E−02	0.937E−02
	0.3	5.215E−06	4.442E−01	0.763E−02	0.511E−01	0.260E−02	0.763E−02
	0.5	2.342E−05	3.891E−01	0.828E−02	0.436E−01	0.220E−02	0.828E−02
	0.7	0.406E−03	3.695E−01	0.137E−01	0.391E−01	0.150E−02	0.137E−01
	0.9	0.158E−01	3.648E−01	0.266E−01	0.355E−01	0.130E−02	0.266E−01
PS-SQP	0.1	3.824E−06	5.933E−01	0.937E−02	0.600E−01	0.440E−02	0.937E−02
	0.3	5.232E−06	4.442E−01	0.763E−01	0.657E−01	0.260E−02	0.763E−02
	0.5	2.341E−05	3.891E−01	0.828E−02	0.699E−01	0.190E−02	0.828E−02
	0.7	0.406E−03	3.695E−01	0.137E−01	0.722E−01	0.150E−02	0.137E−01
	0.9	0.158E−01	3.648E−01	0.266E−01	0.722E−01	0.130E−02	0.266E−01
GA	0.1	1.224E−06	4.256E−01	0.946E−02	0.600E−01	0.360E−02	0.946E−02
	0.3	5.632E−05	4.670E−01	0.103E−01	0.657E−01	0.430E−02	0.103E−01
	0.5	4.856E−05	4.981E−01	0.132E−01	0.699E−01	0.490E−02	0.132E−01
	0.7	0.634E−01	5.172E−01	0.201E−01	0.722E−01	0.520E−02	0.201E−01
	0.9	0.201E−02	5.224E−01	0.338E−01	0.722E−01	0.520E−02	0.338E−01
GA-SQP	0.1	6.86E−09	2.680E−05	7.920E−06	5.42E−06	2.940E−11	7.920E−06
	0.3	0.293E−03	0.387E−03	0.338E−03	1.45E−05	2.110E−10	3.380E−04
	0.5	0.241E−02	0.246E−02	0.244E−03	8.49E−06	7.200E−11	2.440E−03
	0.7	0.879E−02	0.883E−02	0.881E−02	7.79E−06	6.060E−11	8.820E−03
	0.9	0.223E−01	0.223E−01	0.223E−01	7.23E−06	5.220E−11	2.240E−02

## Comparison through statistics

**Fig. 5:**
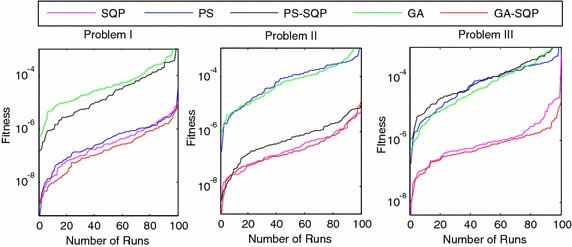
Fitness of 100 independent runs taken by five different algorithms

**Fig. 6:**
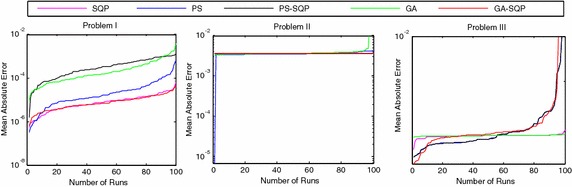
A graphical representation for 100 independent runs and MSE

**Fig. 7:**
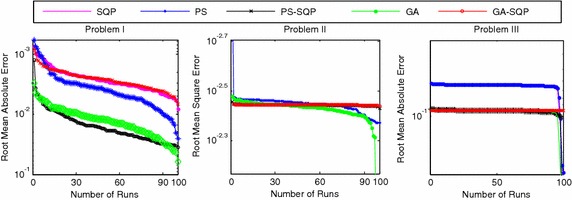
Fitness of 100 independent runs and RMAE for five different algorithms

**Fig. 8:**
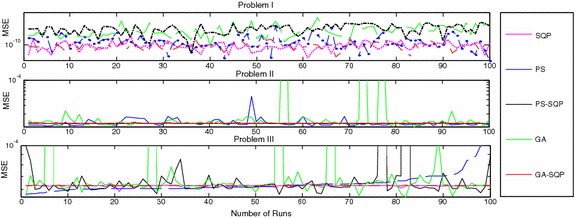
MSE against number of independent runs for each algorithm problem
- The selection of appropriate number of neurons in the construction of ANN models has a significant role in the accuracy and complexity of the algorithms. The performance measuring indices bases on MAE, MSE, and RMAE are used to determine/evaluate the most suitable number of neurons in the proposed ANN models.
- The histogram studies which show the relative frequency of obtaining performance indices values in certain range. Behavior of proposed methodologies through histogram plots are analyzed for all three problems

We present the comparative studies on the basis of following:

- The comparative studies for the given five proposed artificial intelligence solvers are presented for solving LEEs in terms of fitness, mean square error (MSE) and root mean absolute error (RMAE) which is plotted in Figs. 5, 6 and 7, respectively. Furthermore, MSE results of scattered data are shown in Fig. 8.

**Fig. 9:**
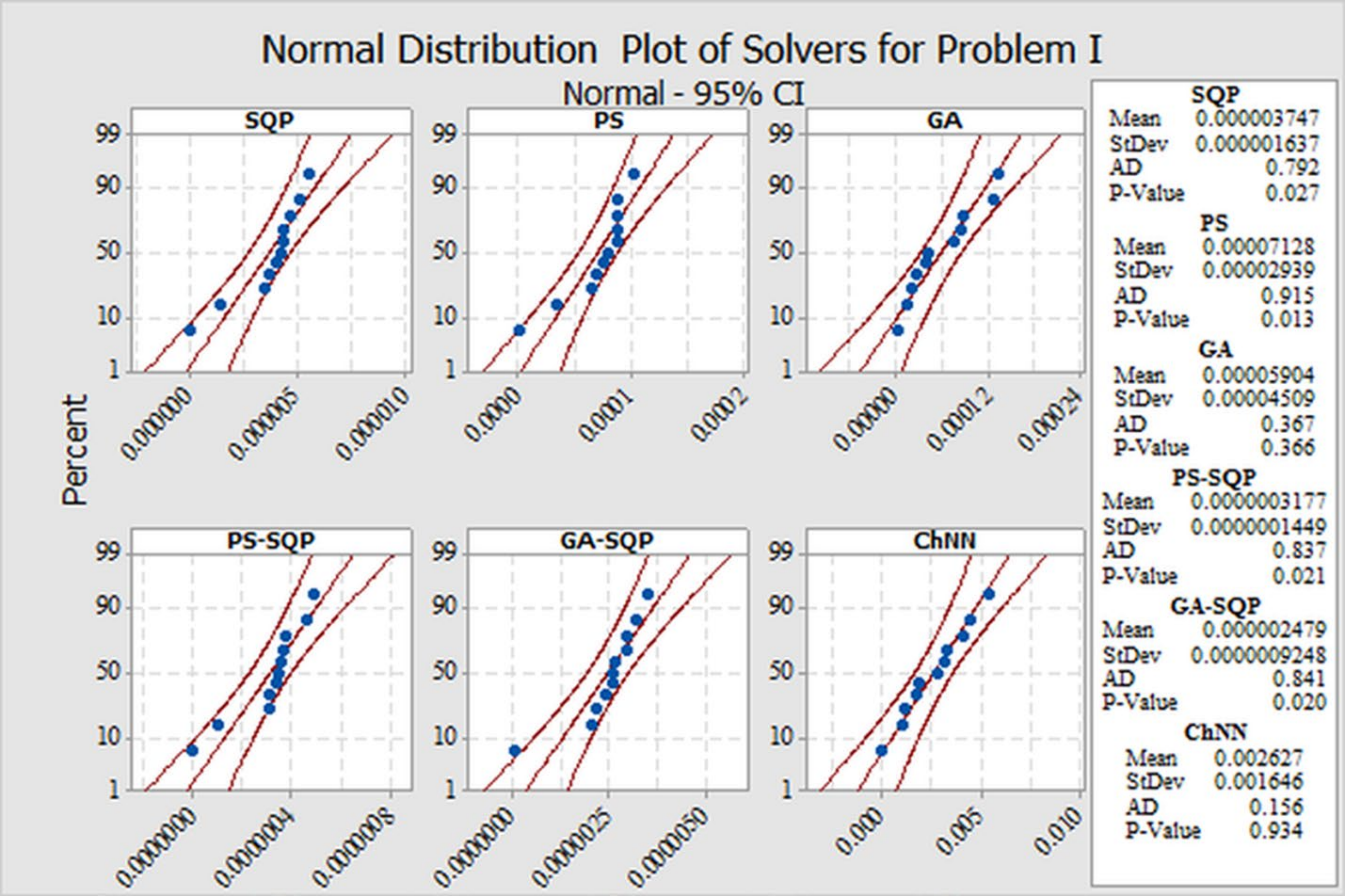
Normal distribution plot for Problem I for different results

**Fig. 10:**
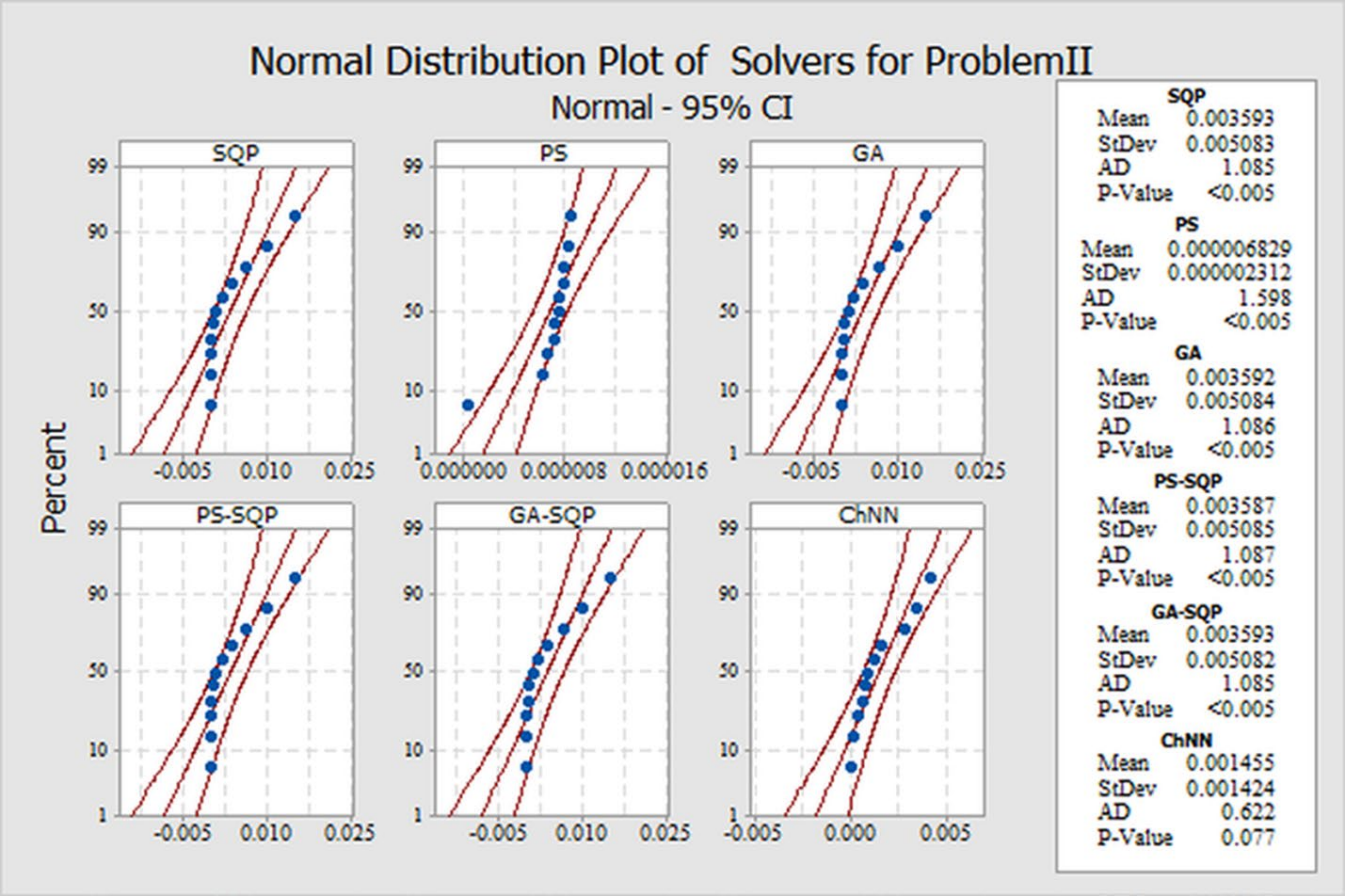
Normal distribution plot for Problem II for different results

**Fig. 11:**
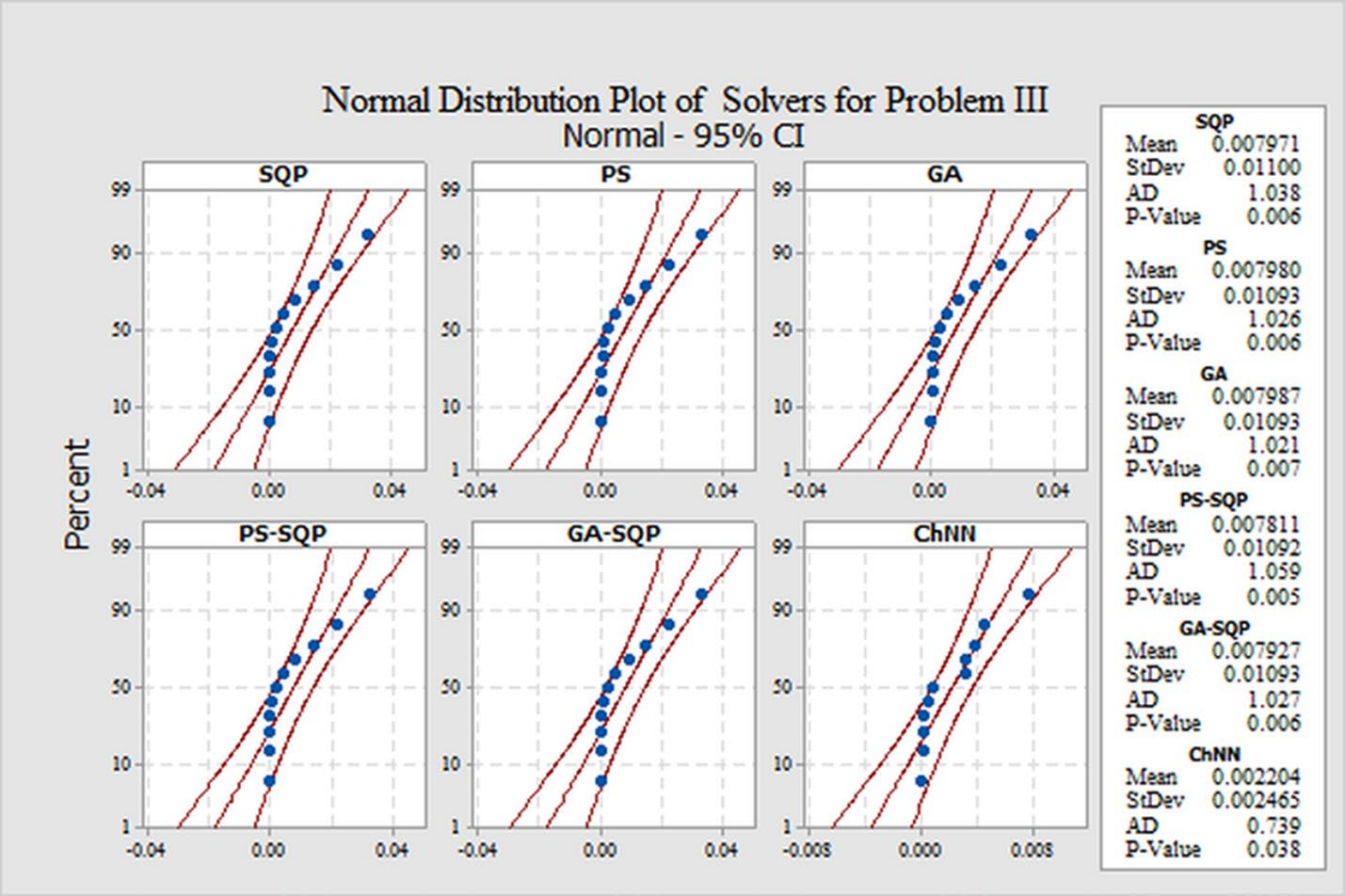
Normal distribution plot for Problem III for different results

**Fig. 12:**
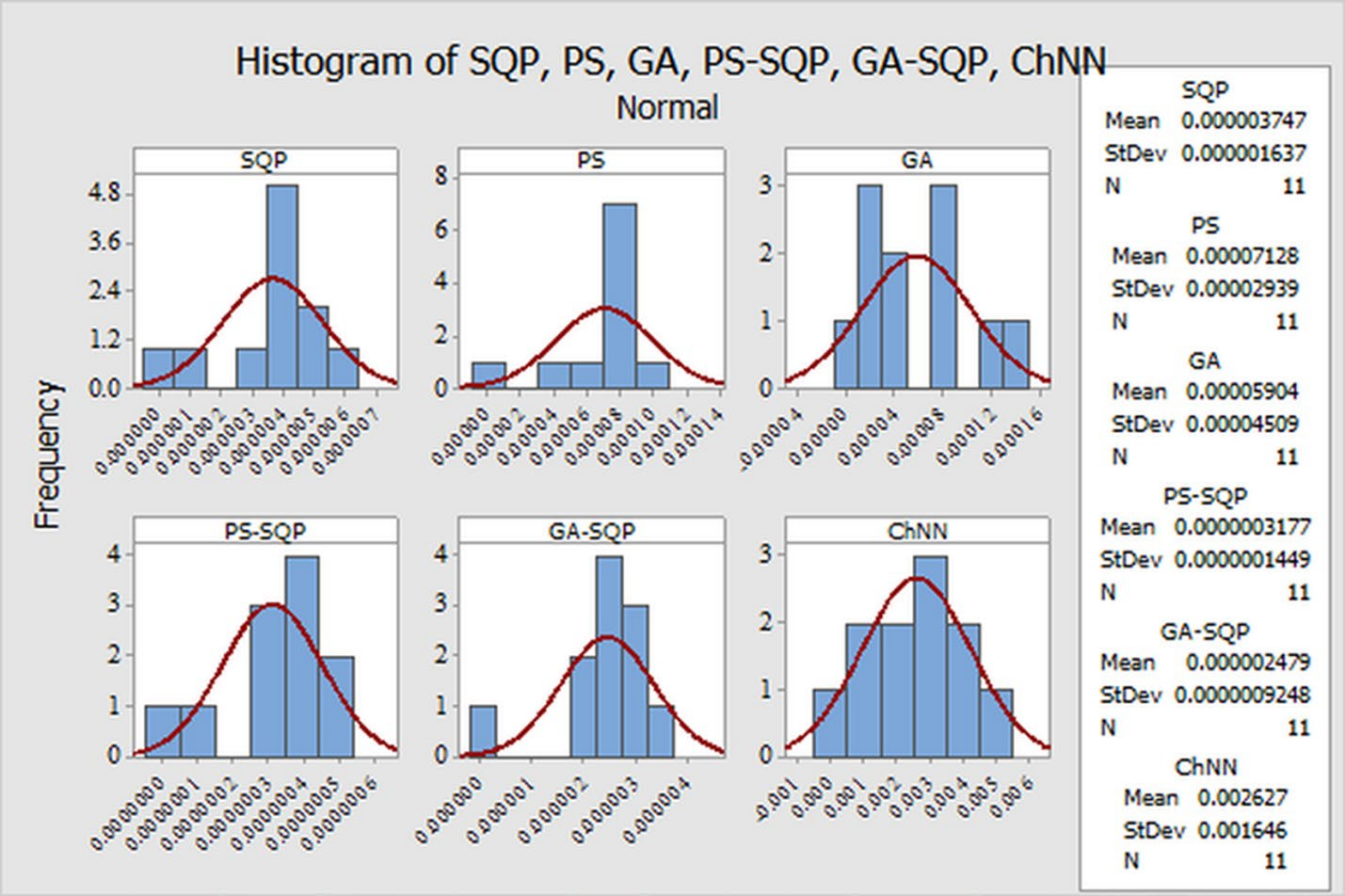
Normality curve fitting of data for Problem I for different results

**Fig. 13:**
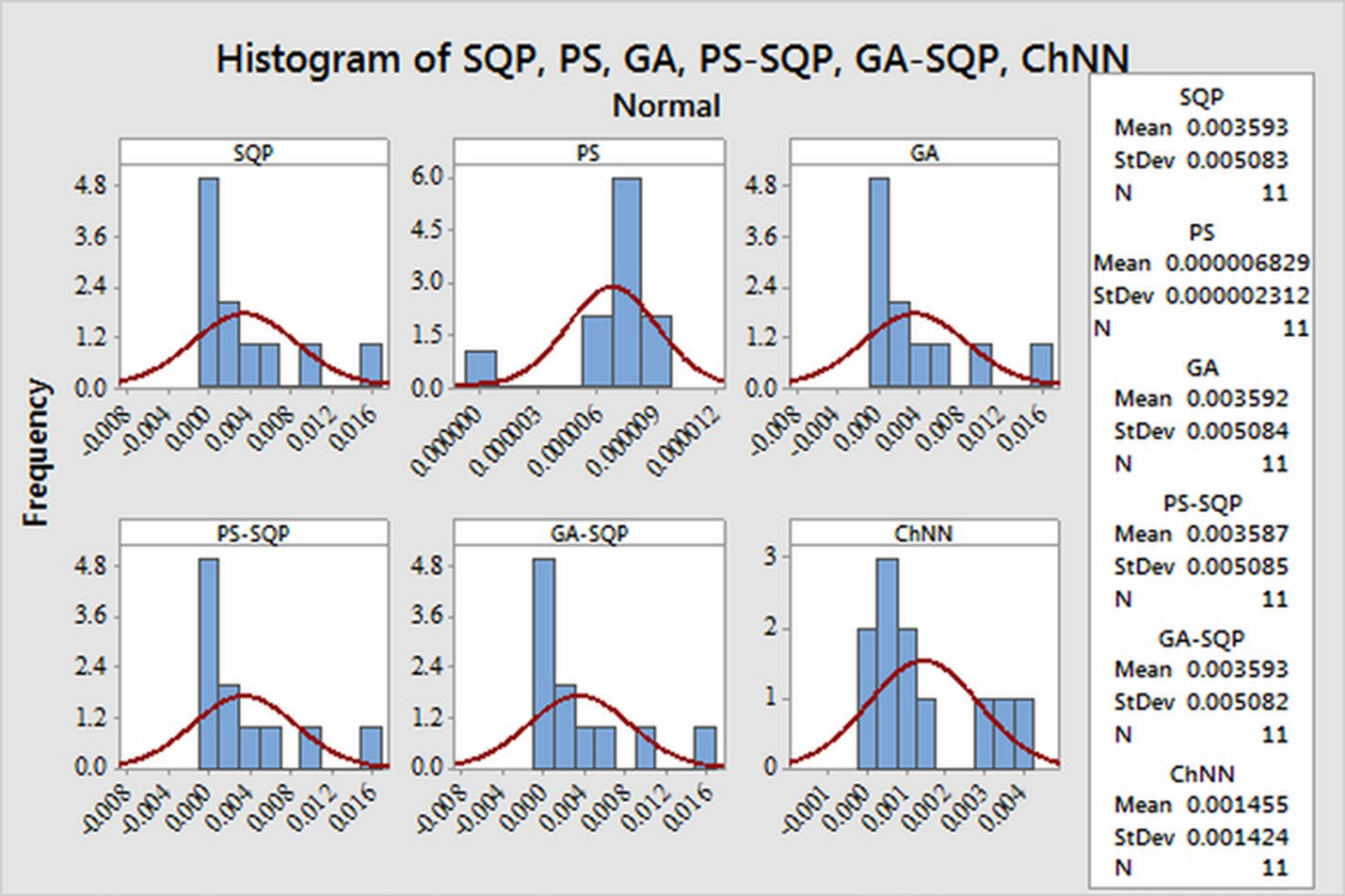
Normality curve fitting of data for Problem II for different results

**Fig. 14:**
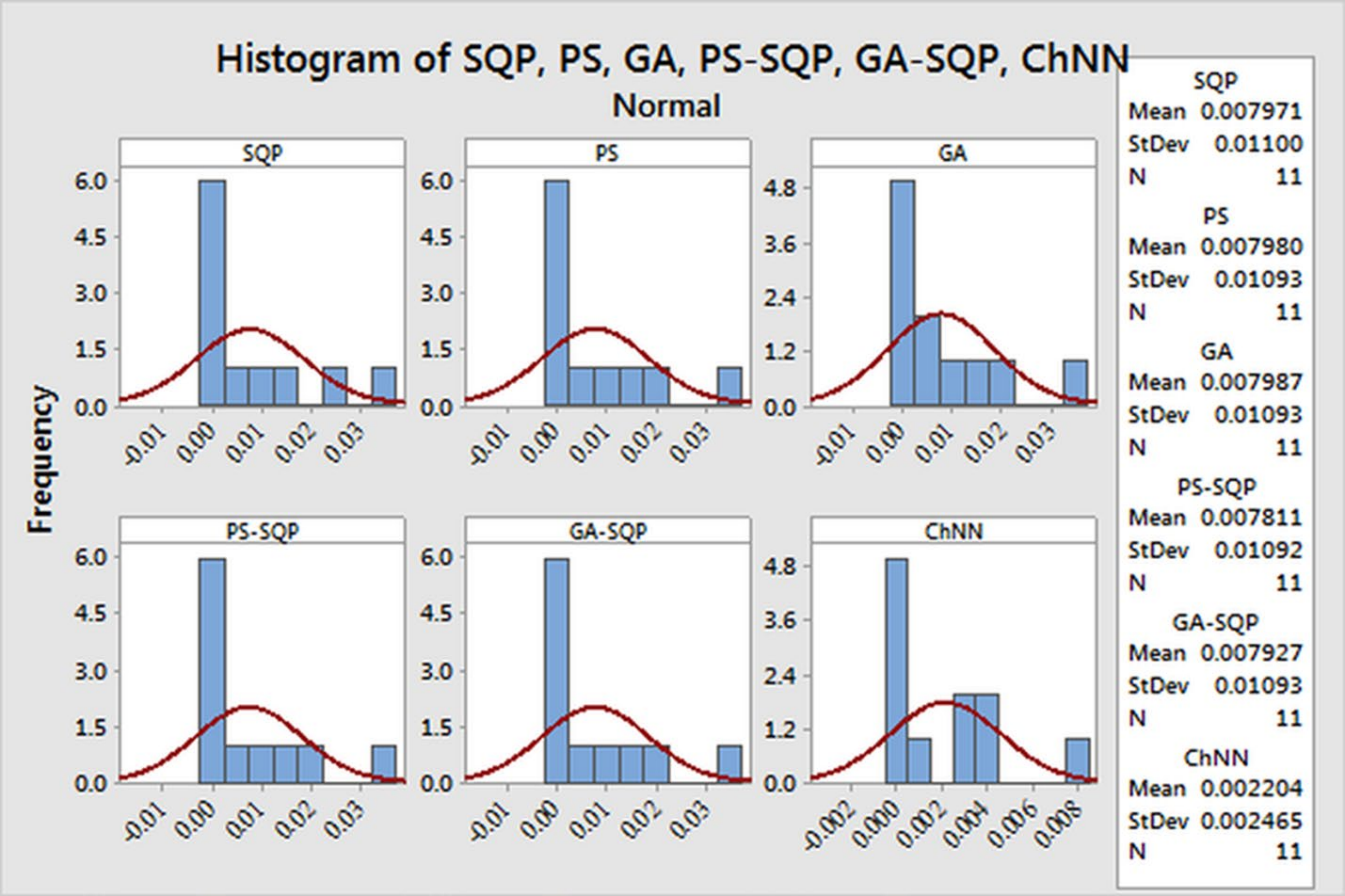
Normality curve fitting of data for Problem II for different results

In order to evaluate small differences, we presented a statistical analysis, particularly fitting of normal distribution based on absolute errors (AEs) of SQP, PS and GA algorithms as shown in Figs. 9, 10 and 11 for problems I, II and III, respectively. The normal curve fitting is used to find how much the normal distribution accurately fits to AEs of our proposed results of algorithms with reported results as presented in Fig. 12. Figure 13 and 14 for problems I, II and III, respectively. In the Figs. 9, 10 and 11 also display 95 % confidence intervals (dotted curves) for the fitted normal distribution.

These confidence levels indicate that the performance of all six methods based on the fitted normal distribution and SQP showed higher accuracy than the other five in Problem I, in Problem II PS showed higher accuracy than the other five. But in the Problem III hybrid technique PS-SQP showed higher accuracy than the other five solvers.

It can be easily observed from these figures that, the result obtained by technique SQP in Problem I. PS in Problem II and hybrid PS-SQP in problem III is better than the results obtained by others algorithms. It is observed that for *N* = 30, our techniques show approximately better results from reported results and obtain the potential to minimize the errors.

## Conclusions

In this research work, a detailed simulation process and statistical analysis of each case with multi-time independent test of each algorithm has been presented. Therefore, on the basis of these runs, we concluded the following important points.

- The best advantage of the solver based on computational techniques with SQP, PS and GA algorithm to represent the approximate solution of Lane–Emden type differential equations as shown in Fig. 4.
- The multi-runs of each algorithm independently provide a strong evidence for the accuracy of the proposed method.
- The problem is still open for future work with the combination of different activation functions like Bessel’s polynomial and B-Polynomial etc.
- The potential area of investigations to exploring in the existing numerical solver to deal with singularity along with strong nonlinear problems like nonlinear Lane–Emden equation based systems.
- In future one may explore in Runge–Kutta numerical methods with adjusted boundary conditions as a promising future research direction in the domain of nonlinear singular systems for effective solution of Lane–Emden equation arising in astrophysics models for which relatively few solvers are available.

## Appendix

The necessary description of the routines used in the simulation studies are given for the ease of reproduction of the results. The software package of Matlab is used, in particularly Matlab optimization toolbox including ‘ga’ and ‘gaoptimset’ for Genetic algorithm, ‘patternsearch’ and ‘psoptimset’ for pattern search method, and ‘fmincon’ and ‘optimset’ for sequential quadratic programming algorithm. While the necessary coding for artificial neural network modeling of the equation, a good source of references is available in Matlab central file exchange server.
